# Using Ecological Momentary Assessment in Testing the Effectiveness of an Alcohol Intervention: A Two-Arm Parallel Group Randomized Controlled Trial

**DOI:** 10.1371/journal.pone.0078436

**Published:** 2013-11-05

**Authors:** Carmen V. Voogt, Emmanuel Kuntsche, Marloes Kleinjan, Evelien A. P. Poelen, Lex A. C. J. Lemmers, Rutger C. M. E. Engels

**Affiliations:** 1 Behavioural Science Institute, Radboud University, Nijmegen, The Netherlands; 2 Addiction Switzerland, Research Institute, Lausanne, Switzerland; 3 Trimbos Institute, Netherlands Institute of Mental Health and Addiction, Utrecht, The Netherlands; Catholic University of Sacred Heart of Rome, Italy

## Abstract

**Background:**

Alcohol consumption of college students has a fluctuating nature, which might impact the measurement of intervention effects. By using 25 follow-up time-points, this study tested whether intervention effects are robust or might vary over time.

**Methods:**

Data were used from a two-arm parallel group randomized controlled trial applying ecological momentary assessment (EMA) with 30 data time-points in total. Students between 18 and 24 years old who reported heavy drinking in the past six months and who were ready to change their alcohol consumption were randomly assigned to the experimental (*n* = 456: web-based brief alcohol intervention) and control condition (*n* = 451: no intervention). Outcome measures were weekly alcohol consumption, frequency of binge drinking, and heavy drinking status.

**Results:**

According to the intention-to-treat principle, regression analyses revealed that intervention effects on alcohol consumption varied when exploring multiple follow-up time-points. Intervention effects were found for a) weekly alcohol consumption at 1, 2, 3, 4, and 7 weeks follow-up, b) frequency of binge drinking at 1, 2, 7, and 12 weeks follow-up, and c) heavy drinking status at 1, 2, 7, and 16 weeks follow-up.

**Conclusions:**

This research showed that the commonly used one and six month follow-up time-points are relatively arbitrary and not using EMA might bring forth erroneous conclusions on the effectiveness of interventions. Therefore, future trials in alcohol prevention research and beyond are encouraged to apply EMA when assessing outcome measures and intervention effectiveness.

**Trial registration:**

Netherlands Trial Register NTR2665

## Introduction

In the last decades, various interventions have been developed to reduce the global burden resulting from health-threatening behaviours, such as excessive alcohol consumption [1]. Randomized controlled trials (RCTs) are considered the gold standard and have been increasingly used to evaluate intervention effectiveness [2]. In alcohol prevention research, efficacy trials commonly report significant differences between conditions in outcome measures, assuming that these effects are attributed solely to the intervention without considering possible measurement artefacts (e.g., time-frame problems [3]). This is troublesome due to major shortcomings related to the way in which the outcome measures are typically assessed and the number of follow-up time-points that are commonly used to test intervention effectiveness.

First, retrospective assessment methods with relative long reference periods (e.g., 30-days or longer) at baseline and follow-up are used [4], thereby increasing the likelihood of recall bias. Precise recall of alcohol consumption decreases after two or three days due to memory deficits [5]-[8] leading to an underreporting of alcohol intake. Moreover, participants are often asked to report the “average” quantity and frequency of alcohol consumption in a “usual” reference period. This further decreases the accuracy of reported alcohol intake [9] since community events (e.g., holidays) and personal events (e.g., birthdays) are likely to be not- or underreported even though they are associated with elevated risk of excessive drinking [10]. Moreover, since recall bias was found to vary as a function of how much alcohol individuals consume [8], it might also differ between individuals who received an intervention versus individuals in the control condition. In the most extreme case, it is possible that reported intervention effectiveness might be simply due to differences in recall bias between the intervention and the control condition. Recall bias threatens the internal validity and thus the credibility of study findings of trials, which is especially worrisome in the light of the usually small to medium effect sizes reported in intervention studies [11], [12]. 

Second, to overcome the problem of recall bias, one can consider using short reference periods that facilitate recall by asking participants to report the exact number, size, and type of alcohol beverage consumed on each day in the past week. Yet, caution is warranted when short reference periods are used and when effects are measured with few follow-up time-points. An important disadvantage of this approach is that it does not consider the fluctuating nature of alcohol consumption among individuals. Moreover, it is unlikely that short reference periods with few follow-up time-points capture important drinking events, such as end of academic year parties, New Year’s Eve, or birthday celebrations [13], [14]. This could lead to biased conclusions that would be based on the selection of (arbitrary) days or weeks as follow-up time-points to test intervention effectiveness. This is especially problematic when the intervention is assumed to cause changes in alcohol consumption but the baseline assessment is completed during high-risk drinking periods (e.g., starting weeks of semester) and follow-ups are completed during low-risk drinking periods (e.g., exams weeks).

Although there are exceptions [15], the majority of trials in alcohol prevention research have used relative long reference periods (i.e., 30 days or longer) to assess outcome measures and few follow-up time-points (i.e., four or less) to test intervention effectiveness [16]-[18], thereby ignoring the fluctuating nature of alcohol consumption among individuals. The current study deals with the evaluation of a web-based brief alcohol intervention for young adults. In line with the aims outlined in the trial study protocol [19], we reported the main outcomes on measurements after one and six months follow-up using the CONSORT Statement and the impact of the intervention on the development of alcohol consumption over time elsewhere [20]. The current study employs post-hoc analyses in which we consider the fluctuating nature of alcohol consumption among individuals. We used short reference periods (i.e., one week) with multiple follow-up time-points (i.e., 25) to test whether intervention effects are robust or vary over time using an ecological momentary assessment (EMA) approach [21]. This, since it is simply not sufficient to use few follow-up time-points (e.g., one and six months follow-up only) to examine the impact of a given alcohol intervention due to the fluctuating nature of alcohol consumption among individuals. The “ecological” aspect of EMA implies that data are collected in real-life settings at strategically selected moments in time. The “momentary” aspect of EMA involves that the assessment of the behaviour under study focuses on participants’ current or recent state. Besides, EMA is characterized by repeated and multiple assessments over time and often used equivalent to experience sampling methods (ESM) [21], [22]. Week-to-week variations in the effects of a web-based brief alcohol intervention were assessed by using 25 follow-up time-points across six months. We analysed the treatment outcome at each follow-up time-point separately; as if these would be 25 independent scenarios with a pre-test and post-test design. Effects are considered robust if the 25 different scenarios come to a similar conclusion about intervention effects. However, due to the fluctuating nature of alcohol consumption among young adults [10], [13], [14], we expected that the effects of the web-based brief alcohol intervention vary across the 25 follow-up time-points. If our hypothesis is correct, these findings would have important implications for the number of follow-up time-points needed for testing intervention effectiveness in future trials in alcohol prevention research and beyond.

## Methods

For the trial study protocol; see www.ncbi.nlm.nih.gov/pmc/articles/PMC3096588/ [19]. The CONSORT checklist for this trial is available as supporting information; see Checklist S1.

### Ethics Statement

The Ethical Committee of the Faculty of Social Sciences of Radboud University Nijmegen approved the study [19].

### Participants and Procedure

**Figure 1:**
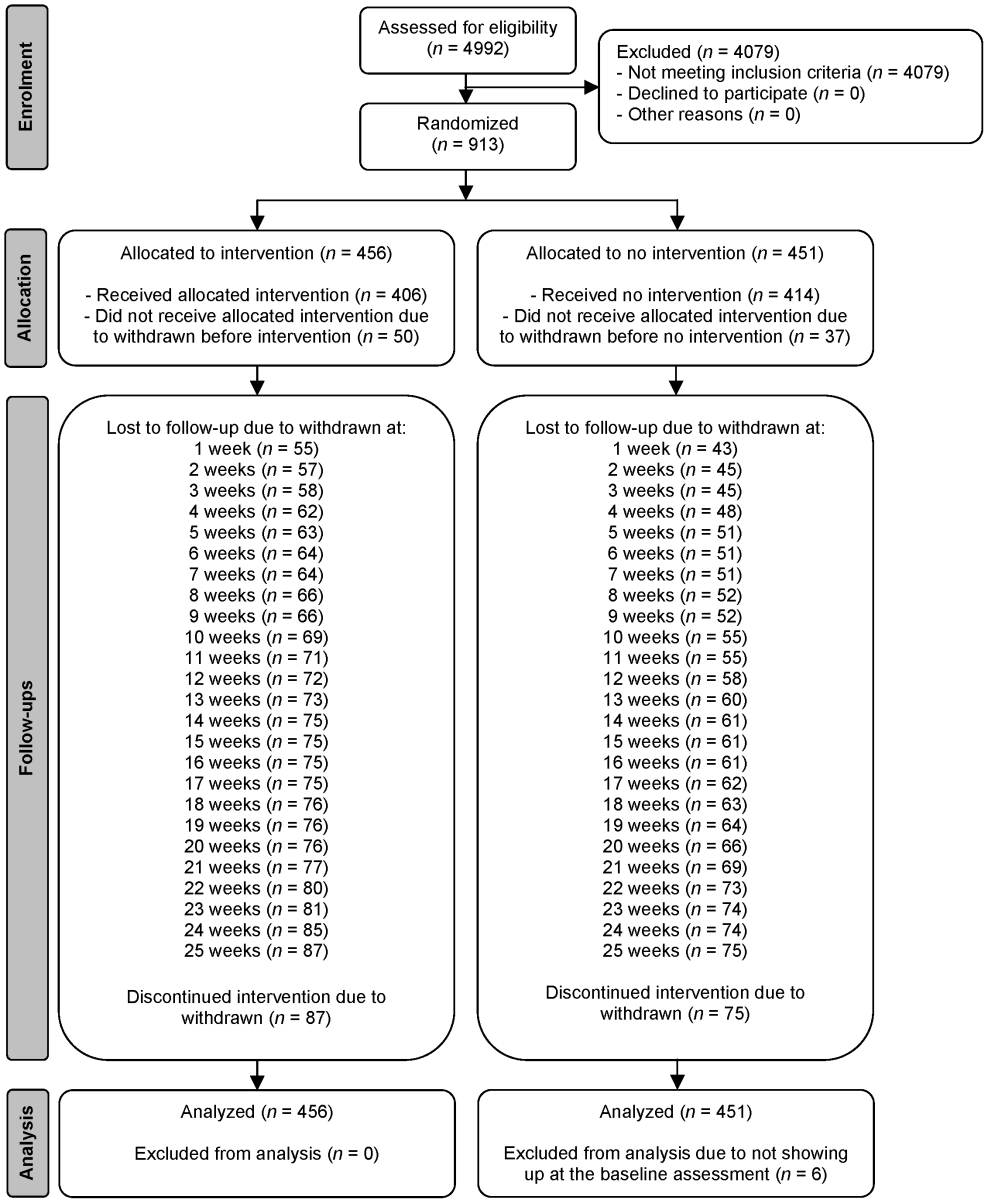
Flow diagram following Consolidated Standards of Reporting Trials (CONSORT) guidelines.

The current study used data from a two-arm parallel group RCT applying an EMA approach with 30 data time-points. From September until December 2010, participants were recruited at Higher Professional Education (HBO) institutions and universities in the Netherlands via distributing flyers. Students between 18 and 24 years old who reported heavy drinking in the past six months, were ready to change their alcohol consumption, had daily access to the Internet, and signed an informed consent form were included in the study. Students reporting a score of 20 or higher on the Alcohol Use Disorders Identification Test (AUDIT: [23]) and/or receiving treatment for alcohol-related problems were excluded from the study and advised to seek treatment since the intervention was developed for the reduction of heavy drinking and not the reduction of problem drinking. A sample size of 908 participants was required given an anticipated dropout rate of 30% after randomization to detect an increase in the percentage of participants adhering to low-risk drinking guidelines after one month of 42% in the experimental condition versus 31% in the control condition [24] with a two-sided 5% significance level and a power of 80%. Students who met the inclusion criteria were randomly assigned to the experimental condition and control condition by an independent researcher of the Behavioural Science Institute (see Figure 1). Randomization occurred centrally using a blocked randomization scheme (block size 4) and was stratified by sex before the baseline assessment in January 2011 [19].

Participants’ drinking patterns were measured at pre-tests and post-tests using EMA. After four EMA pre-test measurements in January, participants in the experimental condition received access to the web-based brief alcohol intervention while participants in the control condition received no intervention. Immediately after the intervention in the first week of February, participants in both conditions received the fifth EMA-measurement, which was the first follow-up time-point. One week after the intervention, all participants received weekly EMA-measurements for six months from February until August. In total, 30 EMA-measurements or data time-points were employed. The web survey software application “Perseus Survey Solutions 6” was used to collect participants’ answers. Participants received a monetary incentive of 100 euro after completing at least 28 out of 30 surveys. This trial is registered in the Netherlands Trial Register (no. NTR2665).

### Interventions

The single session web-based brief alcohol intervention entitled “What Do You Drink” (WDYD) is designed to detect and reduce alcohol consumption among heavy drinking young adults. Completion time of the intervention was approximately 20 minutes. The Intervention Mapping protocol [25] was used to develop the intervention. Content is based on the principles of Motivational Interviewing [26] and parts of the I-Change model [27], in which knowledge, social norms, and self-efficacy are included as the most changeable determinants of behavioral change. Part one of the WDYD intervention contains a screening procedure and personalized feedback based on the screening outcomes (i.e., personal drinking profile). Part two of the WDYD intervention focuses on goal-setting, action planning, and reinforcing drinking refusal self-efficacy through providing tips to maintain drinking goals in situations in which it is hard to resist alcohol. A full description of the WDYD intervention is given elsewhere [19]. Participants in the control condition received no intervention.

### Outcome Measures

Primary outcome measures were weekly alcohol consumption, frequency of binge drinking, and heavy drinking status assessed at baseline and 25 weekly follow-ups by using an EMA approach. EMA is generic term encompassing various research methods that utilize repeated measurements to assess participant’s current or recent states or behaviours in real-life settings at strategically selected moments in time EMA [21].

**Weekly Alcohol Consumption.** Weekly alcohol consumption, defined as the mean number of glasses of standard alcohol units consumed in the past seven days, was assessed using the Dutch version of the Alcohol Weekly Recall [28]. Participants could indicate retrospectively the exact number, size, and type of alcohol beverage they consumed on each day in the past seven days. Standardized responses were assured by providing an overview of standard units for various beverages with one unit representing ten grams of ethanol. Participants who scored three standard deviations above the sample mean of weekly alcohol consumption were given that value in order to retain outliers in the analyses (resulting range 0 to 109) [29]. Weekly alcohol consumption was analyzed as a continuous outcome measure.

**Frequency of Binge Drinking.** Binge drinking frequency was operationalized as the number of days in the past week in which females and males had drunk five or more glasses of standard alcohol units per occasion [30]. The frequency of binge drinking could be answered on an 8-point Likert scale ranging from (0) “never” to (7) “every day”. Frequency of binge drinking was analyzed as a continuous outcome measure.

**Heavy Drinking Status.** Heavy drinking status was defined as the percentage of participants drinking above the normative limits of the Dutch National Health Council for low-risk drinking, which sets a maximum of 14 or 21 glasses of standard alcohol units per week for females and males, respectively [31]. Heavy drinking status was dichotomized into 0 =  “no heavy drinker” and 1 =  “heavy drinker”.

### Analyses

Data were analyzed conforming to the intent-to-treat (ITT) principle and the completers-only framework. The predictive mean matching method (MMS) was employed to impute missing data in SPSS 19. Twenty imputed datasets were evaluated for statistical significance with *p* = 0.05 as criterion by averaging the results (i.e., pooling). A completers-only framework was conducted on participants who completed baseline and all 25 follow-up time-points. The regressions analyses in the completers-only framework were handled in the same way as in the ITT analysis and thus adjusted for baseline measures of the outcome measures. To examine week-to-week variations in the effects of the web-based brief alcohol intervention across six months, regression analyses were conducted for every single follow-up time-point. We utilized linear regression analyses for the outcome measures of weekly alcohol consumption and frequency of binge drinking, whereas we applied logistic regression analyses for heavy drinking status. The Bonferroni correction for multiple testing [32] was not applied since the aim of the study was to test whether intervention effects are robust or vary over time while considering the fluctuating nature of alcohol consumption. Therefore, it was needed to analyze the alcohol outcomes at each follow-up time-point separately; as if these would be 25 independent scenarios with a pre-test-post-test design. For all data points, regression coefficients *(B),* standard errors *(SE)* were reported for weekly alcohol consumption and frequency of binge drinking, whereas odd ratios *(OR)* and 95% confidence intervals *(CI)* were reported for the likelihood to have a consumption above the heavy drinking status threshold. The three outcome measures were regressed on condition (i.e., 0* = *control and 1  =  intervention) while adjusting for baseline measures of the outcome variables.

The four EMA pre-test measures were aggregated into a baseline average while the fifth EMA-measurement conducted immediately after the intervention was not included in the analyses, since participants reported on the drinking behaviour over the past week, thereby making it impossible to observe direct intervention effects. Non-completers (*n* = 162) did not differ from completers (*n* = 745) with respect to the demographic characteristics (i.e., sex: χ*^2^* =  0.34 (*df* = 1), *p* = 0.56, age: t(902) =  −0.25, *p  = * 0.80, education: χ*^2^* =  1.88 (*df* = 1), *p = *0.17, and readiness to change alcohol consumption: χ*^2^* =  0.12 (*df* = 1), *p = *0.73) and outcome measures (i.e., weekly alcohol consumption: t(903) =  0.32, *p = *0.75, frequency of binge drinking: t(903) =  −0.57, *p = *0.57, and heavy drinking status: χ*^2^* =  0.12 (*df* = 1), *p = *0.73) at baseline. The distribution of the missing values indicated that 9.6% of the 907 participants (*n = *87) did not complete the EMA-study and that 8.3% of the 907 participants (*n = *75) nearly completed the survey (missing one or two out of 30 EMA-measurements).

## Results

### Participant Flow

The participant flow throughout the study is presented in Figure 1. Of the 4,992 students who completed the screening survey, 913 met the inclusion criteria of the study. Six students were excluded from the sample because they did not fill in the baseline survey. Of the 907 students, 456 (50.3%) were allocated to the experimental condition and 451 (49.7%) to the control condition. In total, 745 completed the baseline assessment and all 25 EMA follow-ups. The attrition rate at 25 EMA follow-ups was 17.9% (*n = *162) due to withdrawn and was distributed equally between the two conditions (χ*^2^* =  0.927 (*df* = 1), *p = *0.34). The analyses were performed over 907 participants by original assigned conditions.

### Baseline Characteristics

Table 1 depicts demographic characteristics and outcome measures of 907 participants. The average age was 20.8 (*SD = *1.7), 60.3% of the participants were male, 73.5% received university training, and 21.4% were motivated to reduce alcohol consumption in the near future. The screening survey was administered between September and December 2010, whereas the baseline assessment was administered in January 2011, which might explain the lower rates of participant’s readiness to change alcohol consumption at baseline. At baseline, mean weekly alcohol consumption was 21.9 (*SD = *13.5) alcohol units, frequency of binge drinking was 1.8 (*SD = *1.0) times per week, and 51.2% were classified as heavy drinkers. There were no significant differences (*p* > 0.05) between conditions on any of the baseline variables.

**Table 1:** Demographic characteristics and outcome measures at baseline (*N = *907).

	Intervention (*n = *456)	Control (*n = *451)	Total sample (*N = *907)
Male, %	60.3	60.3	60.3
Age, mean (SD)	20.9 (1.7)	20.8 (1.7)	20.8 (1.7)
Education: attending HBO, %	26.8	26.2	26.5
Education: attending university, %	73.2	73.8	73.5
Contemplation stage^a^, %	20.4	22.4	21.4
Weekly alcohol consumption^b^, mean (SD)	22.0 (13.0)	21.9 (14.0)	21.9 (13.5)
Frequency of binge drinking^c^, mean (SD)	1.8 (1.0)	1.7 (1.1)	1.8 (1.0)
Heavy drinking status^d^, %	52.0	50.3	51.2

*Note.* All differences between conditions were non-significant (*p* > 0.05). SD: standard deviation. HBO: Higher Professional Education. ^a^ Readiness to change alcohol consumption was assessed through one item asking the participants which statement applied best to them. Participants selecting “I want to reduce drinking alcohol within the upcoming six months” or “I want to reduce drinking alcohol within the upcoming month” were considered to be in the contemplation stage of change, meaning that they were willing to reduce their alcohol consumption in the near future. ^b^ The mean number of glasses of standard alcohol units consumed in the past seven days. ^c^ The number of days in the past week drinking five or more glasses of standard alcohol units per occasion. ^d^ Drinking > 14 and > 21 glasses of standard units of alcohol per week for females and males, respectively. One standard alcohol unit represents ten grams of ethanol.

### Effect of the Intervention

**Figure 2:**
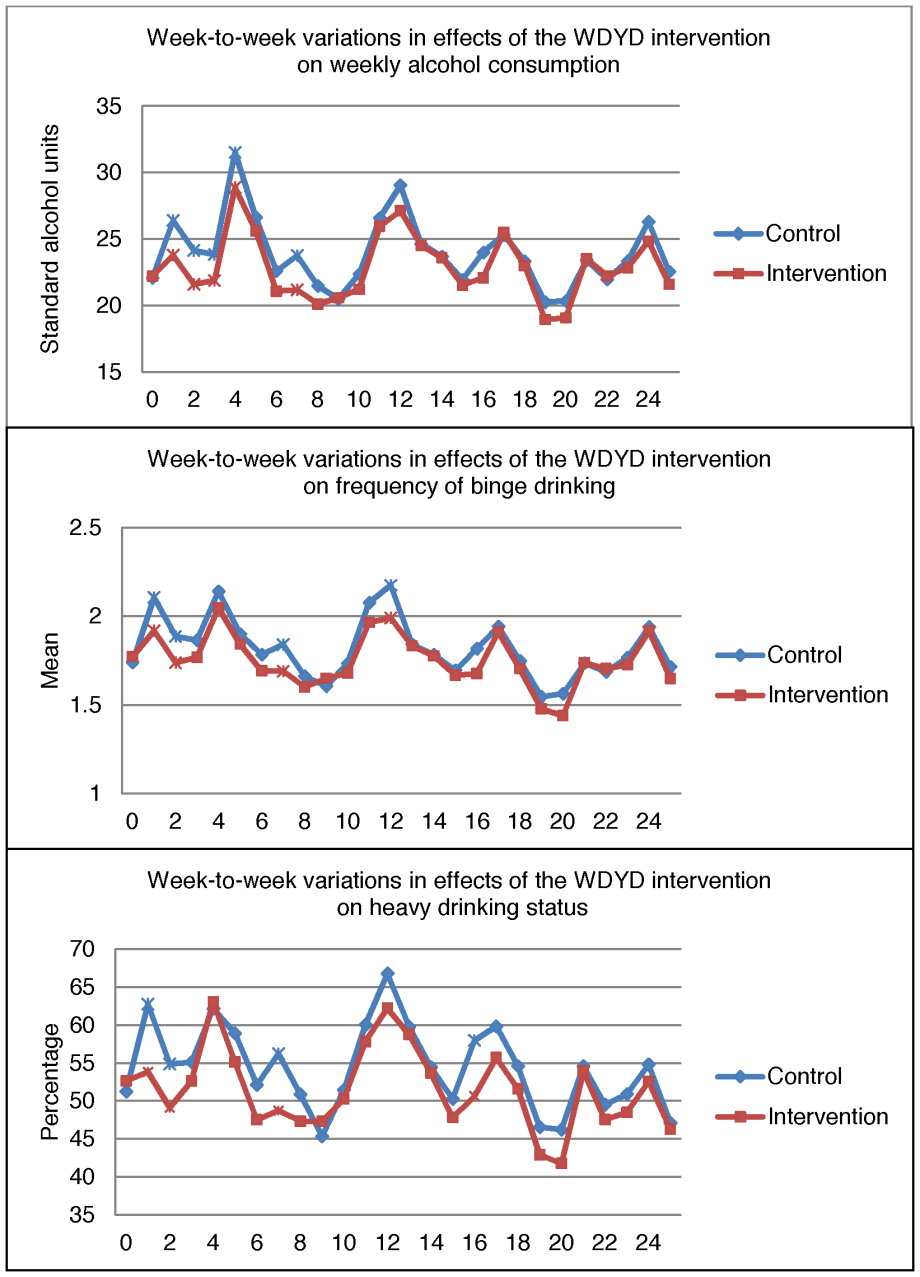
Week-to-week variations in effects of the WDYD intervention on weekly alcohol consumption, frequency of binge drinking, and heavy drinking status at 25 EMA follow-ups by condition. *N* *= *907. *significant.

**Weekly Alcohol Consumption.** The intervention significantly reduced weekly alcohol consumption in the experimental condition relative to the control condition at 1, 2, 3, 4, and 7 weeks follow-ups, respectively (see Table 2 and Figure 2). In both conditions, weekly alcohol consumption varied over time. In the experimental condition, mean weekly alcohol consumption ranged from 18.9 (*SD = *16.4) alcohol units at 19 weeks follow-up to 28.9 (*SD = *22.9) at 4 weeks follow-up compared to 20.2 (*SD = *17.3) and 31.5 (*SD = *26.3) alcohol units in the control condition. The 4^th^ EMA follow-up time-point coincided with carnival, a four-day event celebrated in February before spring in the southern provinces in the Netherlands, and it is associated with excessive drinking. These results were replicated in the completers-only analyses (findings can be obtained from the first author upon request). 

**Table 2:** Weekly alcohol consumption at 25 EMA follow-ups by condition. *N = *907.

	Intervention (*n = *456)		Control (*n = *451)			
Follow-up in weeks	M	SD	M	SD	B	SE
1	23.8	18.6	26.4	19.5	–2.74**	1.03
2	21.6	16.7	24.1	19.2	–2.67**	0.93
3	21.9	16.6	23.8	19.3	–2.07*	0.94
4	28.9	22.9	31.5	26.3	–2.78*	1.43
5	25.6	20.5	26.6	20.2	–1.13	1.19
6	21.1	16.6	22.6	18.1	–1.61	0.96
7	21.2	16.3	23.7	18.2	–2.68**	0.97
8	20.1	16.5	21.5	17.0	–1.46	0.91
9	20.6	16.9	20.5	17.5	0.00	0.95
10	21.2	16.9	22.4	18.3	–1.23	1.01
11	25.9	19.8	26.6	21.0	–0.75	1.21
12	27.1	20.0	29.0	20.0	–2.02	1.18
13	24.5	18.7	24.6	18.5	–0.22	1.04
14	23.6	18.7	23.7	19.9	–0.18	1.10
15	21.5	17.6	21.9	17.7	–0.51	1.03
16	21.1	18.5	24.0	19.0	–2.01	1.09
17	25.5	20.2	25.3	19.3	0.09	1.15
18	23.0	19.3	23.3	18.9	–0.43	1.12
19	18.9	16.4	20.2	17.3	–1.39	0.98
20	19.1	17.3	20.4	17.9	–1.39	1.02
21	23.5	18.8	23.4	18.9	–0.11	1.10
22	22.2	19.6	22.0	19.4	0.16	1.14
23	22.8	21.8	23.6	21.5	–0.61	1.34
24	24.8	23.2	26.3	24.2	–1.58	1.50
25	21.6	20.6	22.5	19.8	–1.04	1.28

*Note.* * *p* <.05. ** *p* <.01. *** *p* <.001. M: mean. SD: standard deviation. B: unstandardized regression coefficient. SE: standard error. One standard alcohol unit represents ten grams of ethanol.

**Frequency of Binge Drinking.** Analyses showed that participants in the experimental condition reported significantly fewer binge drinking occasions compared to participants in the control condition at 1, 2, 7, and 12 weeks follow-up (Table 3 and Figure 2). In the experimental condition, frequency of binge drinking ranged from 1.4 (*SD = *1.4) at 20 weeks follow-up to 2.0 at 4 (*SD = *1.5), 11 (*SD = *1.6), and 12 (*SD = *1.4) weeks follow-up. Frequency of binge drinking ranged from 1.5 (*SD = *1.4) at 19 weeks follow-up to 2.2 (*SD = *1.4) at 12 weeks follow-up in the control condition. The completers-only analyses revealed that the intervention was effective at 12, 16, and 20 weeks follow-up (findings can be obtained from the first author upon request). Frequency of binge drinking was not entirely normally distributed. Therefore, we re-estimated the results by maximum likelihood estimation with robust standard errors (MLR) in Mplus. However, the results were consistent with the ones presented here (findings of the tables can be obtained from the first author upon request). 

**Table 3:** Frequency of binge drinking at 25 EMA follow-ups by condition. *N = *907.

	Intervention (n* = *456)		Control (n* = *451)			
Follow-up in weeks	M	SD	M	SD	B	SE
1	1.9	1.5	2.1	1.5	–0.21*	0.09
2	1.7	1.4	1.9	1.5	–0.17*	0.08
3	1.8	1.4	1.9	1.5	–0.12	0.09
4	2.0	1.5	2.1	1.6	–0.11	0.09
5	1.8	1.4	1.9	1.4	–0.08	0.09
6	1.7	1.4	1.8	1.5	–0.11	0.09
7	1.7	1.4	1.8	1.5	–0.17*	0.09
8	1.6	1.4	1.7	1.4	–0.08	0.08
9	1.6	1.4	1.6	1.5	0.02	0.09
10	1.7	1.4	1.7	1.5	–0.08	0.09
11	2.0	1.6	2.1	1.6	–0.13	0.10
12	2.0	1.4	2.2	1.4	–0.20*	0.09
13	1.8	1.4	1.8	1.4	–0.03	0.09
14	1.8	1.4	1.8	1.6	–0.03	0.09
15	1.7	1.4	1.7	1.4	–0.05	0.09
16	1.7	1.4	1.8	1.5	–0.16	0.09
17	1.9	1.5	1.9	1.5	–0.05	0.09
18	1.7	1.4	1.7	1.5	–0.06	0.09
19	1.5	1.4	1.5	1.4	–0.09	0.09
20	1.4	1.4	1.6	1.4	–0.14	0.09
21	1.7	1.3	1.7	1.4	–0.02	0.09
22	1.7	1.6	1.7	1.6	–0.00	0.10
23	1.7	1.7	1.8	1.7	–0.06	0.11
24	1.9	1.8	1.9	1.9	–0.04	0.12
25	1.6	1.7	1.7	1.7	–0.09	0.12

*Note.* * *p* <.05. ** *p* <.01. *** *p* <.001. M: mean. SD: standard deviation. B: unstandardized regression coefficient. SE: standard error.

**Heavy Drinking Status.** At 1, 2, 7, and 16 weeks follow-ups, a significantly higher number of participants in the experimental condition drank within the normative limits of the Dutch National Health Council for low-risk drinking compared to those in the control condition (Table 4 and Figure 2). Heavy drinking was highest (63.0%) at 4 weeks follow-up and lowest (40.8%) at 20 weeks follow-up for participants in the experimental condition. For those in the control condition, heavy drinking was highest at 12 weeks follow-up (66.8%) and lowest (45.4%) at 9 weeks follow-up. All findings were replicated under completers-only analyses (findings can be obtained from the first author upon request).

**Table 4:** Percentage of participants drinking above the normative limits of the Dutch guidelines for low-risk drinking (drinking > 14 or > 21 (female/male) glasses of standard units of alcohol per week) at 25 EMA follow-ups by condition. *N = *907.

	Intervention (*n = *456)	Control (*n = *451)		
Follow-up in weeks	%	%	OR	95% CI
1	53.8	62.7	0.62**	(0.46 to 0.85)
2	49.1	54.9	0.73*	(0.54 to 0.99)
3	52.6	55.1	0.86	(0.64 to 1.16)
4	63.0	62.2	1.02	(0.76 to 1.38)
5	55.1	58.9	0.82	(0.62 to 1.09)
6	47.5	52.1	0.79	(0.59 to 1.06)
7	48.8	56.2	0.68*	(0.51 to 0.92)
8	47.3	50.9	0.82	(0.61 to 1.10)
9	47.3	45.4	1.07	(0.80 to 1.44)
10	50.3	51.5	0.92	(0.68 to 1.26)
11	57.8	60.1	0.89	(0.66 to 1.18)
12	62.2	66.8	0.78	(0.57 to 1.05)
13	58.7	59.8	0.93	(0.69 to 1.25)
14	53.7	54.5	0.94	(0.70 to 1.27)
15	47.8	50.3	0.87	(0.65 to 1.17)
16	50.7	58.0	0.69*	(0.52 to 0.93)
17	55.7	59.8	0.80	(0.60 to 1.08)
18	51.6	54.6	0.86	(0.65 to 1.14)
19	42.9	46.6	0.83	(0.62 to 1.10)
20	41.8	46.2	0.79	(0.59 to 1.07)
21	53.9	54.6	0.95	(0.71 to 1.27)
22	47.5	49.5	0.90	(0.68 to 1.20)
23	48.5	50.9	0.89	(0.67 to 1.18)
24	52.5	54.8	0.89	(0.68 to 1.18)
25	46.3	47.1	0.95	(0.71 to 1.27)

*Note.* * *p* <.05. ** *p* <.01. OR: odds ratios. CI: confidence interval. One standard alcohol unit represents ten grams of ethanol.

## Discussion

The current study examined week-to-week variations in the effects of a web-based brief alcohol intervention to test whether intervention effects are robust over time or vary due to the fluctuating nature of alcohol consumption. Data were used from a trial that applied an EMA approach with 25 follow-up time-points conducted across six months. As expected, the effects of the web-based brief alcohol intervention on the outcome measures varied across the 25 follow-up time-points. Additionally, intervention effects varied across the different outcome measures in terms of both the frequency and timing of the effects. The fluctuating pattern of intervention effects over time in this study raises questions with respect to the credibility of findings reported in former trials in alcohol prevention research. One might inquire about the degree to which findings reported in earlier trials are reliable when a) 30-day or longer reference periods were used to assess outcome measures, b) four or less (arbitrary) follow-up time-points were used to assess intervention effectiveness, and c) the fluctuating nature of alcohol consumption among individuals was not considered. Caution should thus be exercised when interpreting findings of trials in alcohol prevention research since intervention effects seem to vary from week to week across outcome measures, which makes that conclusions regarding intervention effectiveness differ depending on the selection of follow-up time-points. In our case, no intervention effect was found at two months follow-up, but one week prior to two months follow-up there was an effect. Former trials on intervention effectiveness in alcohol prevention research might have found significant main effects when selecting other follow-up time-points. Moreover, significant main effects might even become insignificant when using short reference periods with more precise recall, especially if small effect sizes were reported. Our findings have potentially important implications for the testing of outcome measures and the number of follow-up time-points needed to assess intervention effectiveness in future trials in alcohol prevention research and beyond.

### Advantages of EMA

The use of short reference periods with multiple follow-up time-points by means of an EMA approach has rarely been used to assess outcome measures and to test intervention effectiveness in alcohol prevention research. Nonetheless, EMA can overcome shortcomings related to traditional methods of assessing outcomes measures and intervention effectiveness. First, EMA-measurements can generate ecological valid outcome measures of individuals’ alcohol consumption over time since they cover relatively short reference periods, thereby enabling a reduction in memory deficits and recall bias [21]. Improved recall of alcohol consumption can be further enhanced by asking individuals to report retrospectively the exact number, size, and type of alcohol beverage they consumed on each day in the past seven days (e.g., previous Sunday, previous Saturday, etc.) instead of asking them to indicate the “average” number in the past week. Additionally, because EMA outcomes measures are refined and sensitive to change, they might alleviate sample size requirements, making EMA-studies less difficult and less expensive to conduct [33]. Moreover, EMA allows determining whether intervention effects are robust or varying over time. Finally, type I and type II errors can be reduced by aggregating the means of the outcome measures across multiple time-points, thereby generating an overall intervention effect. The reduction of statistical errors results in more reliable outcome measures and a higher precision in measuring intervention effectiveness. Overall, measuring intervention effects by means of an EMA approach will enhance our understanding of how intervention effectiveness develops over time, which can help determine the time at which the intervention effects have levelled off and “booster sessions” (re-exposure to the intervention) are needed to strengthen and/or extend intervention effects.

### Future Directions

The advantages of EMA justify the importance of adopting this method more widely in future trials to measure the effectiveness of alcohol interventions. It might also be beneficial to use EMA to increase the precision of measuring the effectiveness of interventions in research beyond alcohol prevention (e.g., depression, bulimia nervosa), especially when recall bias is present, the outcome measures under the investigation have a high variability across time, and few follow-up time-points are used to assess intervention effectiveness. Nevertheless, to confirm that intervention effects vary when multiple time-points are explored; replication of our findings is needed. Also, future studies are advised to investigate populations other than heavy drinking students, since the reported effects might be less evident in populations with more stable drinking patterns, such as problem drinkers. In addition, the current trial used an inactive treatment in the control condition and did not adjust for participants’ expectations that can affect the outcomes of the trial. Future trials should measure expectations of treatment benefit and the extent to which participants perceive the treatment in the control condition to be as credible as the treatment in the experimental condition to determine whether the conditions are significantly different with regard to this non-specific treatment effect [34]. Besides, as in other trials, EMA consists of self-report measures which presents methodological concerns that should be considered [21]. Also, EMA imposes a higher participant response burden compared to traditional trials with few follow-up time-points, possibly reducing compliance since participants need to devote time, effort, and skills to complete the EMA-study. To facilitate participants’ compliance, investigators should give a briefing about the study procedure before the study onset, use short and well-conducted surveys, and offer monetary incentives after study completion. With a high retention rate of 82.1%, especially compared to traditional trials delivering web-based interventions [35], our trial indicates the feasibility of conducting an EMA study within a RCT context. Furthermore, reactivity or the potential that observed changes in certain behaviours are affected by the act of assessing might occur by employing EMA. Although research has shown that participants’ reactivity can reduce alcohol outcome measures when using traditional assessments methods [36]–[38], evidence that EMA stimulates significant reactivity is limited [21].

## Conclusions

By means of an ecological momentary assessment approach with 25 follow-up measures, this study showed that intervention effects vary over time. The intervention was mainly effective on the short term, which provides important information for implementation purposes, such as the timing of booster sessions. It further showed that the commonly used one and six month follow-up time-points are relatively arbitrary and not using EMA might bring forth erroneous conclusions on the effectiveness of interventions. Besides, EMA can maximize ecological validity, minimize recall bias, and takes into account the fluctuating nature of individuals’ behaviour over time. Therefore, future trials in alcohol prevention research and beyond are encouraged to apply EMA when assessing outcome measures and intervention effectiveness.

## Supporting Information

Checklist S1 **CONSORT Checklist.**
(DOCX)
